# Natural Reward Experience Alters AMPA and NMDA Receptor Distribution and Function in the Nucleus Accumbens

**DOI:** 10.1371/journal.pone.0034700

**Published:** 2012-04-18

**Authors:** Kyle K. Pitchers, Susanne Schmid, Andrea R. Di Sebastiano, Xu Wang, Steven R. Laviolette, Michael N. Lehman, Lique M. Coolen

**Affiliations:** 1 Departments of Anatomy and Cell Biology, Schulich School of Medicine and Dentistry, The University of Western Ontario, London, Ontario, Canada; 2 Departments of Molecular and Integrative Physiology, University of Michigan, Ann Arbor, Michigan, United States of America; 3 Department of Psychology, University of Michigan, Ann Arbor, Michigan, United States of America; Institut National de la Santé et de la Recherche Médicale, France

## Abstract

Natural reward and drugs of abuse converge upon the mesolimbic system which mediates motivation and reward behaviors. Drugs induce neural adaptations in this system, including transcriptional, morphological, and synaptic changes, which contribute to the development and expression of drug-related memories and addiction. Previously, it has been reported that sexual experience in male rats, a natural reward behavior, induces similar neuroplasticity in the mesolimbic system and affects natural reward and drug-related behavior. The current study determined whether sexual experience causes long-lasting changes in mating, or ionotropic glutamate receptor trafficking or function in the nucleus accumbens (NAc), following 3 different reward abstinence periods: 1 day, 1 week, or 1 month after final mating session. Male Sprague Dawley rats mated during 5 consecutive days (sexual experience) or remained sexually naïve to serve as controls. Sexually experienced males displayed facilitation of initiation and performance of mating at each time point. Next, intracellular and membrane surface expression of N-methyl-D-aspartate (NMDA: NR1 subunit) and α-amino-3-hydroxy-5-methylisoxazole-4-propionate (AMPA: GluA1, GluA2 subunits) receptors in the NAc was determined using a bis(sulfosuccinimidyl)suberate (BS^3^) protein cross-linking assay followed by Western Blot analysis. NR1 expression was increased at 1 day abstinence both at surface and intracellular, but decreased at surface at 1 week of abstinence. GluA2 was increased intracellularly at 1 week and increased at the surface after 1 month of abstinence. Finally, whole-cell patch clamp electrophysiological recordings determined reduced AMPA/NMDA ratio of synaptic currents in NAc shell neurons following stimulation of cortical afferents in sexually experienced males after all reward abstinence periods. Together, these data show that sexual experience causes long-term alterations in glutamate receptor expression and function in the NAc. Although not identical, this sex experience-induced neuroplasticity has similarities to that caused by psychostimulants, suggesting common mechanisms for reinforcement of natural and drug reward.

## Introduction

The mesolimbic system consists of interconnected brain areas, including the ventral tegmental area, medial prefrontal cortex (mPFC), and nucleus accumbens (NAc) [1]. Together, these brain areas mediate naturally rewarding behaviors including feeding [2], [3], [4], [5], drinking [6], maternal behavior [7], social bonding [8], [9] and sexual behavior [10], [11], [12], [13], [14]. Behavioral studies have demonstrated that male rat sexual behavior is rewarding and reinforcing as male rats form a conditioned place preference for copulation [15], [16], [17], develop faster running speeds in T-mazes [18], straight-arm runway [19] or hurdle climbing [20], and perform operant tasks to gain access to sexually receptive females [21], [22]. Moreover, sexual experience causes facilitation of subsequent sexual behavior, including increased sexual motivation and performance [23], and influences expression of conditioned place preference for mating [15]. These behavioral changes suggest the occurrence of natural reward-related learning and memory, which is hypothesized to be mediated by alterations in the mesolimbic system induced by mating behavior experience [11].

In support of this hypothesis, we have previously shown that sexual experience caused an upregulation of deltafosB in the NAc, which in turn, was critical for the facilitation of initiation and performance of sexual behavior following sexual experience [23]. In addition, sexual experience caused an increase in dendritic arborization and number of spines in the NAc [11]. Similar changes in transcription and morphology are induced by psychostimulants [24], [25], [26]. Sexual experience has been shown to alter responsiveness to drugs of abuse, including sensitization to psychostimulant-induced locomotor activity (cross-sensitization) and enhanced psychostimulant reward [10], [11], [27]. It has been demonstrated that a withdrawal or abstinence period following drug exposure is critical for an increased craving for the drug, termed incubation of craving [28]. Likewise, a mating abstinence period following sexual experience is critical for sexual experience-induced enhancement of psychostimulant reward and increased dendritic branching and spines in the NAc [11]. Hence, sexual experience and subsequent abstinence from this natural reward cause long-term alterations in the mesolimbic system that are similar to those induced by psychostimulants, and affect behavioral responses to both natural and drug reward.

Psychostimulants have been reported to induce numerous additional alterations in the NAc, several of which are dependent on a drug abstinence period. These alterations include changes in glutamate receptor trafficking and function [29], [30]. Repeated cocaine followed by a long abstinence period (3–7 weeks), causes an increase in surface expression of α-amino-3-hydroxy-5-methylisoxazole-4-propionate (AMPA) receptor subunits and N-methyl-D-aspartate (NMDA) receptor subunits, whereas no changes were observed after a short abstinence period (1 day). Moreover, different subunits of AMPA and NMDA receptors are differentially affected by drug exposure and subsequent abstinence periods, as surface expression of GluA2-lacking AMPARs is up-regulated after prolonged abstinence periods (5–7 weeks) [31], [32]. The PFC provides a major glutamatergic input to the NAc [33] and electrophysiological studies have shown that repeated cocaine causes alterations in AMPA/NMDA ratio in PFC-responding NAc shell neurons and changes in intrinsic excitability of NAc neurons that may be dependent on a drug abstinence period and NAc subregion [34], [35], [36], [37], [38], [39], [40], [41]. Such drug-induced neural plasticity contribute to changes in psychostimulant behavioral sensitization [42], [43], [44], [45] and drug seeking [31], [46], [47].

It is currently unknown whether similar adaptations of glutamate receptor trafficking and function occur following natural reward experience and subsequent abstinence from natural reward. Therefore, the goal of the current study was to test the hypothesis that sexual experience causes changes in AMPA or NMDA receptor trafficking in the NAc and alterations in synaptic strength of PFC-responding NAc shell neurons. In addition, these measures were determined at 3 different times following the final mating session to investigate the effects of an abstinence period from natural reward (1 week or 1 month after final mating) on plasticity in the NAc compared to a no abstinence period (1 day after final mating).

## Results

### Experiment 1: Sex Facilitation

**Figure 1:**
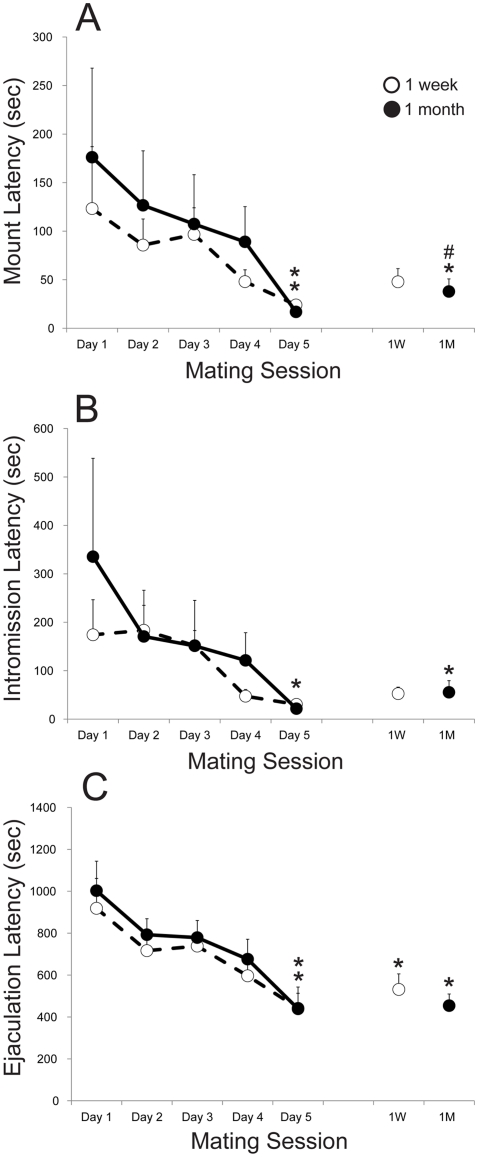
Long-term maintenance of facilitated sexual behavior. Latencies to mount (A), intromission (B), and ejaculation (C) for two experimental groups during 5 daily mating sessions and a final Test either 1 week (white, dashed) or 1 month (black, continuous) following last mating session. Data are expressed as group means (± SEM). * indicates significant difference from Day 1; # indicates significant difference from Day 5.

The objective of experiment 1 was to determine a time course for facilitation of sexual behavior following sexual experience. Previously, facilitation of sexual behavior has been detected up to 1 week after final mating session [23], but it has not been previously investigated if sex experience-induced facilitation of sexual behavior is maintained after a protracted abstinence period. Here, two groups of male rats were tested for long-term expression of facilitation of sexual behavior, either 1 week or 1 month following last mating session. First, both groups showed facilitation of sexual behavior during five daily mating sessions as demonstrated by significantly shorter latencies to mount (Figure 1A; 1 week, p = 0.028; 1 month, p = 0.019), intromission (Figure 1B; 1 month, p = 0.016; trend at 1 week, p = 0.078) and ejaculation (Figure 1C; 1 week, p = 0.016; 1 month, p = 0.008) on day 5 compared to day 1 of sexual experience. Furthermore, facilitation of sexual behavior was maintained both 1 week and 1 month after last mating session, as latencies to mating parameters were shorter on Test day (1 week or 1 month after day 5 of sexual experience) compared to mating day 1 (Figure 1A: mount latency, 1 month, p = 0.016; Figure 1B: intromission latency, 1 month, p = 0.046; Figure 1C: ejaculation latency, 1 week, p = 0.016; 1 month, p = 0.008) and no significant differences existed between mating day 5 and Test day for any behavioral parameter at any time (except for mount latency at 1 month; p = 0.016). Hence, experience-induced facilitation of sexual behavior was maintained during a 1 month period of mating abstinence.

### Experiment 2: Ionotropic Glutamate Receptor Subunit Redistribution/Expression

**Figure 2:**
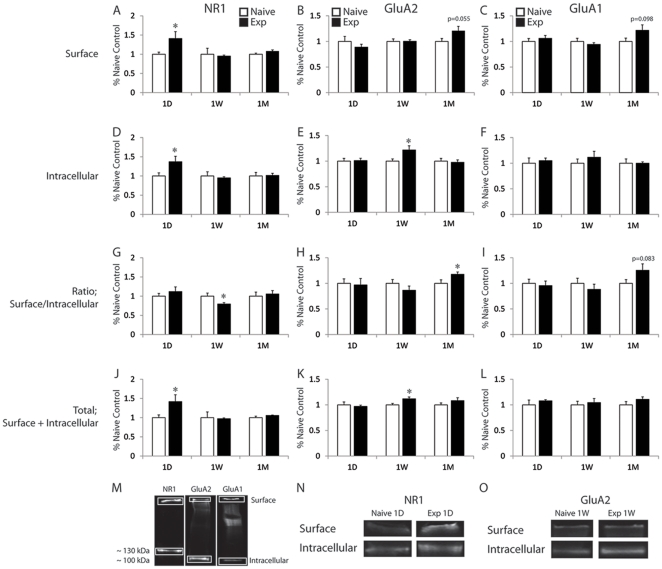
Glutamate receptor subunit expression and distribution in the NAc. Quantitative analysis of expression for NR1 (A, D, G, J), GluA2 (B, E, H, K) and GluA1 (C, F, J, L) for surface (A, B, C), intracellular (D, E, F), surface to intracellular ratios (G, H, I) and total protein levels (J, K, L) in sexually experienced and naïve males at 1 day, 1 week and 1 month following last mating or handling session. Data are expressed as group means (± SEM). * indicates significant difference from sexually naïve males at same time point. Statistical trends are indicated by p-values. Images of representative blots demonstrating surface (>250 kDa) and intracellular bands for NR1 (∼130 kDa), GluA2 (∼100 kDa) and GluA1 (∼106 kDa) (M). Representative images demonstrating increased surface expression of NR1 (N) and intracellular expression of GluA2 (O) at 1D and 1W respectively.

The objective of experiment 2 was to determine the expression of ionotropic glutamate receptor subunits in sexually experienced and naïve males following different mating abstinence periods. AMPAR and NMDAR subunit surface and intracellular pools were quantified using a membrane-impermeant cross-linking reagent (BS^3^), which selectively modified surface-expressed protein enabling distinction from unmodified intracellular proteins using SDS-PAGE and Western Blot analysis. Representative blots illustrating surface (High MW, >250 kDa) and intracellular bands are presented in Figure 2M.

One day following sexual experience, expression of NR1 subunit in surface (S), intracellular (I) and total (S+I) were significantly increased (S, Figure 2A, p = 0.025; I, Figure 2D, p = 0.035; S+I, Figure 2J, p = 0.023) in sexually experienced animals compared to sexually naïve controls (Figure 2N). One week following last mating, there was a significant decrease in surface to intracellular ratio expression of NR1 (S/I ratio; Figure 2G; p = 0.024) without significant changes in surface or intracellular expression, compared to sexually naïve animals. Expression of GluA2 significantly increased one week following last mating, in intracellular and total expression, without changes in surface expression (I, Figure 2E, p = 0.026; S+I, Figure 2K, p = 0.014) in sexually experienced animals compared to naïve (Figure 2O). Whereas, one month following last mating, GluA2 was significantly increased in S/I ratio (Figure 2H; p = 0.046) accompanied with a statistical trend towards an increase in surface expression (Figure 2B; p = 0.055) in sexually experienced animals compared to naïve controls. Changes in GluA1 were not detected at 1 day or 1 week following last mating. After 1 month, GluA1 surface and S/I ratio expression appeared to be increased in sexually experienced animals compared to naïve controls, albeit only statistical trends were detected (S, Figure 2C, p = 0.098; S/I, Figure 2I, p = 0.083). Hence, in summary, initially at 1 day of abstinence, a total increased expression of NR1 was detected, followed by increased intracellular expression of GluA2 after 1 week of abstinence and increased surface expression of GluA2 and GluA1 after 1 month of abstinence (the latter only as a statistical trend).

### Experiment 3: Electrophysiology

**Figure 3:**
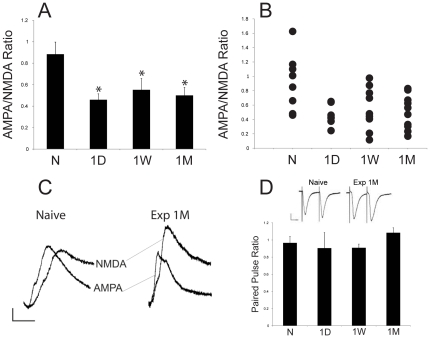
AMPA/NMDA ratios for sexually experienced and naïve males at 1 day, 1 week or 1 month after last mating or handling session. A) Average AMPA/NMDA ratios in NAc shell neurons of sexually naïve (N) and sexually experienced animals, either 1 day (1D), 1 week (1W) or 1 month (1M) after final handling or mating sessions. Data are expressed as group means (± SEM). * indicates significant difference from sexually naïve males. B) Ratio of the peak amplitudes of AMPA and NMDA current traces for each recorded cell. C) Representative AMPA and NMDA traces recorded in medium spiny neurons in the NAc shell of naïve and sexually experienced (1 month after final mating session) animals. Vertical scale bar indicates 20 mV; horizontal scale bar indicates 20 ms. D) Average paired pulse ratios in NAc shell neurons of sexually naïve (N) and sexually experienced animals, either 1 day (1D), 1 week (1W) or 1 month (1M) after final handling or mating sessions. Data are expressed as group means (± SEM). Representative paired pulse current traces of naïve and sexual experienced (1 month after final mating session) animals. Vertical scale bar indicates 50 pA; horizontal scale bar indicates 25 ms.

The objective of experiment 3 was to determine whether sexual experience alters synaptic strength in PFC-responding NAc shell neurons. Synaptic currents were recorded in medium spiny neurons in the NAc shell of sexually experienced and naïve males following stimulation of fibers presumed to derive from the PFC. AMPA/NMDA ratio was significantly reduced in sexually experienced animals 1 day (p = 0.005), 1 week (p = 0.016), and 1 month (p = 0.005) after final mating session compared to the sexually naïve control group (Figure 3A, B, C). In order to determine if there was a change in presynaptic transmitter release probability, paired-pulse ratios were investigated. The magnitude of the facilitation of transmitter release that occurred in response to paired-pulse stimulation did not differ between sexually experienced and naïve animals at any time interval (Figure 3D).

## Discussion

The current study demonstrated that sexual experience causes changes in ionotropic glutamate receptor distribution and function in the NAc of male rats and that some of these changes varied with the length of abstinence period from sexual behavior. Compared to sexually naïve males, sexually experienced males showed a short-term increase in total NR1 subunit expression due to increases in both surface and intracellular receptor pools. GluA2 expression was increased intracellularly and at the surface after 1 week and 1 month of reward abstinence, respectively. Finally, GluA1 expression was not significantly changed at any time. Furthermore, sexually experienced animals had an immediate and long-lasting decreased AMPA/NMDA ratio recorded from PFC-responding NAc shell neurons compared to naïve controls. Our results indicate that glutamate receptor trafficking depends on the length of an abstinence period from sexual behavior, whereas alterations in synaptic strength at synapses formed by prefrontal cortical afferents to the NAc shell do not. These main findings are similar to reports following repeated cocaine in terms of AMPAR subunit increased surface expression after long drug abstinence and an immediate decrease in AMPA/NMDA ratio, but differ in regards to short-term NMDAR subunit expression, and a persistent decrease in AMPA/NMDA ratio.

Sexual behavior is highly rewarding and reinforcing. Thus, as a male rat gains sexual experience, it displays increased sexual motivation and performance, which are demonstrated by shorter latencies to initiate mating and display ejaculation, and increased copulation efficiency [10], [23]. Here, it was confirmed that this facilitation of sexual behavior was present at 1 week following mating, and it was determined that facilitated sexual behavior was maintained for up to 1 month of mating abstinence. The temporal profile of sex experience-induced facilitation of sex behavior corresponds with our previous study showing cross-sensitization to the locomotor-inducing effects of amphetamine when tested at the same mating abstinence periods [11]. Previous studies have implicated the NAc as a mediator of the maintenance of facilitated sexual behavior. Both NAc core and shell neurons are activated by mating or cues-associated with mating [48], and sexual experience results in increased dendritic branching and spines in the NAc core and shell a week after mating [11]. In addition, decreasing the activity of the transcription factor deltaFosB in the NAc via viral vector-mediated gene transfer attenuates experience-induced facilitation of sexual behavior [23], [49]. These findings suggest that the NAc is specifically associated with reinforcement of sexual behavior, and is potentially critical for the cross-sensitized drug effects of sexual experience [23].

The current study demonstrated that sexual experience caused changes in AMPAR synthesis and/or trafficking that were dependent on the length of the sex abstinence period. First, an increase in intracellular expression of GluA2 expression was evident at 1 week, potentially indicating increased GluA2 receptor synthesis and/or endocytosis. Then, an increased GluA2 surface/intracellular ratio (largely due to increase at surface) was detected at 1 month after last mating, suggesting redistribution of receptors to the cell surface. Changes in AMPAR trafficking following cocaine exposure, were also dependent on the length of the drug abstinence period [50]. In general, cell surface and synaptosomal GluA1 and GluA2/3 levels are increased after a week of abstinence from cocaine and are maintained at elevated levels for up to 3 weeks after last cocaine injection [51], [52], [53], [54], [55]. After a more prolonged period of drug abstinence (5 weeks), GluA1 surface expression remained elevated and there was a small decrease in GluA2 surface/intracellular ratio, and hence GluA2-lacking AMPARs were upregulated [31], [56]. This increased expression of GluA2-lacking receptors at the surface was detected after prolonged withdrawal (5–7 weeks) from extended-access cocaine self-administration, but not following prolonged withdrawal from non-contingent cocaine [32]. Moreover, blockade of GluA2-lacking AMPAR prevented drug-seeking [31], [57]. The changes in AMPA receptor trafficking following sexual experience and abstinence vary slightly from those induced by cocaine. In terms of increased GluA1 surface expression, the present data indicate a modest increase in GluA1 receptors at the membrane surface that failed to reach statistical significance at 1 month after sex experience, and thus suggesting that GluA1 surface expression may require longer abstinence periods for further up-regulation, similar to the changes induced by cocaine after 5 weeks of abstinence [31]. Furthermore, the present data differ in terms of GluA2 which underwent a small decrease after 5 or more weeks without cocaine, while sexual experience and 1 month of abstinence resulted in increased GluA2 surface expression. We hypothesize that AMPA receptor up-regulation in NAc may be critical for the long-term effects of sexual experience on subsequent reward behavior and increased amphetamine reward [11] following the prolonged abstinence periods tested in the current study. In contrast, alterations in AMPA receptor expression or trafficking are not critical for the short-term effects of sexual experience on reward behavior. In support, numerous reports have demonstrated that altered AMPAR transmission is not necessary for drug-induced behavioral sensitization to psychostimulants (for review [30]). Sensitization and cross-sensitization have been observed after 1 day of reward abstinence without changes in AMPAR upregulation [11], [53]. Moreover, behavioral sensitization has been demonstrated with repeated exposure to the psychostimulant amphetamine, which has not been associated with any changes in AMPAR transmission [50], [53], [58].

The role of NMDARs has been much less studied than AMPARs in regards to behavior sensitization and receptor trafficking. A non-selective NMDAR antagonist (MK-801) prevented the development of locomotor sensitization with cocaine or amphetamine, but failed to block expression of this sensitization [59], [60]. The role of NMDAR in sexual behavior has been minimally examined through systemic or intra-medial preoptic area administration of MK-801, which impaired sex behavior in naïve and experienced male rats [61], [62], [63]. NMDAR also mediate other natural rewards as NMDAR antagonists decreased food intake in baboons [64] and enhanced food craving in rats [65]. NMDAR expression in the NAc is altered by cocaine exposure, and longer (3 weeks) but not short (1 day) abstinence periods from repeated cocaine increased the expression of NMDAR subunits (NR1, NR2A, NR2B) [55], [60]. The current results demonstrate that sexual experience caused an increase in total NR1 expression at 1 day due to increased levels in both the surface and intracellular receptor pools, in addition to decreased surface/intracellular ratio after a 1 week abstinence period. Hence, we hypothesize that an initial increase in NMDAR transmission may be critical for the short-term effects of sexual experience on subsequent reward behavior.

Moreover, the short-term increase in NR1 subunit following sexual experience may be indicative of sex experience-induced silent synapse formation. Huang et al [66] demonstrated that repeated cocaine generated silent synapses in the NAc shell, in which the post-synaptic recruitment of new NMDAR was key. Silent glutamatergic synapses express functional NMDAR-mediated currents in absence of AMPAR mediated currents [67], [68], [69]. It has been shown that previous cocaine exposure can generate silent synapses throughout the brain and that these newly generated synapses provide a substrate for subsequent experience [66], [70]. NMDAR are composed of a least one NR1 subunit in combination with one or more NR2 subunits (A–D). NR1 is required for the formation of functional channels; hence changes in NR1 expression may provide an index for changes in the number of functional NMDA receptors. Repeated cocaine drives the insertion of NR1 and NR2B- containing NMDARs and generates silent synapses in the NAc shell. These NR2B-containing silent synapses were inhibited during cocaine administration which attenuated subsequent locomotor sensitization with cocaine [71]. The number of silent synapses decreased after several days of cocaine abstinence whereas locomotor sensitization persisted, suggesting premature synapses may engage in the formation of new plastic circuits that can be altered (potentially by reward abstinence) to mediate these persistent behaviors. Thus, we hypothesize that up-regulation of NR1 total expression shortly following sexual experience may be due to the increase number of NMDAR in newly formed silent synapses. Furthermore, sexual experience followed by protracted abstinence may result in decreased silent synapses, explaining both the decreased NR1 surface/intracellular ratio and the increase in AMPAR subunits with prolonged abstinence periods, as synapses are unsilenced.

The current findings that sexual experience and reward abstinence periods caused alterations in glutamate receptor trafficking and expression suggested alterations in the synaptic strength at excitatory synapses in the NAc. Therefore, an electrophysiology procedure based on a previous study by Thomas and coworkers [72] was utilized to study glutamate receptor function in NAc shell neurons following the same abstinence periods shown to result in sensitized sex and drug behavior, and glutamate receptor redistribution. Similar to previous studies following cocaine exposure, plasticity was determined in NAc shell synapses that were responsive to PFC input. PFC is well established to play a key role in compulsive behavior and drug abuse [73], [74]. Likewise, the PFC is critical for development or expression of compulsive sexual behavior, as PFC lesions cause maladaptive seeking of sexual behavior in male rats [75]. The current data showed that sexual experience caused an immediate and long-lasting decrease in AMPA/NMDA ratio in the PFC-responding NAc shell neurons.

Previously, it was hypothesized that the primary difference between natural and drug reward is that changes in AMPA/NMDA ratio following natural reward are temporary and will dissipate with time whereas drug-induced changes will persist [76]. This hypothesis was based on findings that cocaine, but not food or sucrose induced a long-lasting increase AMPA/NMDA ratio in the VTA [76]. In contrast, the current study demonstrates that sexual experience did indeed cause a long-lasting change in AMPA/NMDA ratio in the NAc. However, the sex-induced NAc shell plasticity differs from that following cocaine exposure, which have been shown to be associated with a bidirectional change in AMPAR mediated excitability. Following cocaine exposure and one day of drug abstinence, a reduction in AMPA/NMDA ratios [72] and depression in firing capacity [34] was found, similar to the results obtained for short-term effects of sexual experience. However, after a 14 days abstinence period from cocaine, NAc shell neurons have increased AMPA/NMDA ratios, suggesting that abstinence from cocaine enhanced synaptic strength in the NAc [54]. Hence, cocaine exposure results in a bidirectional change in synaptic plasticity with reduced AMPA-mediated responses shortly after drug exposure, but increased AMPAR-mediated excitability with protracted drug abstinence. In contrast, protracted abstinence from sexual experience resulted in decreased AMPAR-mediated excitability in NAc shell, even though sensitization of locomotor activity and reward associated with amphetamine are observed during those abstinence periods [11]. However, other reports have shown decreased intrinsic membrane excitability in both the core and shell at short and long abstinence period following cocaine exposure [35], [36], [37], [38], [39], [40], [41], showing a lack of bidirectional change, similar to the effects of sexual experience. Moreover, the current results show sexual experience does not cause changes in presynaptic glutamate release probability based on lack of differences between groups for paired pulse ratio. Kalivas and colleagues have demonstrated that repeated cocaine induces changes in mediators of extracellular non-synaptic glutamate pools in the NAc, whether such alterations occur with sexual experience remain to be determined (for review [77]).

It is likely that glutamatergic inputs to the NAc from other brain areas than the PFC play a functional role in the effects of sexual experience on subsequent reward behavior. The NAc receives glutamatergic input from the basolateral amygdala (BLA) and the ventral subiculum of the hippocampus [78]. The BLA plays a role in natural reward-seeking behavior [79], [80], is critical for the association of environmental stimuli with sexual reward [81] and for instrumental behavior to initiate mating [82]. Hence, it is tempting to speculate that sex experience-induced alterations in synaptic strength at BLA-responding synapses as well.

The decreased AMPA/NMDA ratio at PFC synapses in the NAc shell is consistent with the increased expression of NR1 shortly after sexual experience, but inconsistent with increased GluA2 and GluA1 after prolonged abstinence periods. One explanation is that receptor trafficking occurred at synapses receiving glutamatergic input arising from other brain areas besides the PFC. Also, the inclusion of NAc core, in addition to the NAc shell, in the protein samples exclude the possibility to make accurate correlations between the protein expression and synaptic strength data. Nonetheless, the current findings clearly demonstrate that natural reward behavior and subsequent reward abstinence cause synaptic plasticity in the NAc with similarities and differences from those induced by psychostimulants.

In conclusion, sexual experience and subsequent reward abstinence in male rats was shown to cause immediate and long-lasting facilitation in sexual behavior, similar to the effects of sexual experience on sensitization to the locomotor inducing effects of amphetamine [11], and was associated with an immediate and long-lasting decrease in AMPA/NMDA ratios in synapses in NAc shell. Moreover, sexual experience caused a fast upregulation of NMDAR and slowly developing redistribution of AMPAR to the surface of NAc neurons. Hence, similar to the effects of psychostimulants, natural reward behavior can cause long-lasting alterations in glutamate receptor expression, trafficking, and excitability. However, protracted abstinence from sexual experience, unlike that following cocaine, resulted in persisted decrease in AMPA/NMDA ratio in the NAc shell. A number of testable hypotheses for potential underlying contributing factors for this discrepancy were proposed, including alterations of BLA-responding synapses, following natural reward experience. These data once again show both similarities and differences between the long-term consequences of natural and drug reward exposure and may contribute to a better understanding of how drugs may act on this natural reward pathway.

## Methods

### Ethics Statement

All procedures were approved by the Animal Care and Use Committee of the University of Western Ontario and conformed to Canadian Council on Animal Care guidelines involving vertebrate animals in research.

### Animals

Adult male Sprague Dawley rats (experiment 1 and 2:10–12 weeks; experiments 3, 8–10 weeks at time of onset of experiments) were obtained from Charles River Laboratories (Senneville, QC, Canada). Same-sex animals were pair-housed in Plexiglas cages with a tunnel tube in a temperature-regulated room maintained on a 12/12 hr light dark cycle with food and water available *ad libitum* except during behavioral testing. Stimulus females (210–220 grams) for mating sessions received a subcutaneous implant containing 5% estradiol benzoate and 95% cholesterol following bilateral ovariectomy. Sexual receptivity was induced by administration of 500 µg progesterone in 0.1 mL sesame oil approximately 4 hours before testing.

### Sexual Experience

All male rats were sexually naïve prior to the onset of the experiments. Mating sessions occurred during the early dark phase (between 2–6 hours after onset of the dark period) under dim red illumination. During each mating session male rats were allowed to copulate to ejaculation or until 1 hour, and parameters for sexual behavior were recorded, including: mount latency (ML; time from introduction of female until first mount), intromission latency (IL; time from introduction of female until first mount with vaginal penetration), and ejaculation latency (EL; time from first intromission to ejaculation) [83]. Sexually naïve controls were handled, housed in the same rooms as the mating animals and thus exposed to the same levels of noise, general disturbance and distant smells of estrous females, but were not allowed to interact or mate with receptive females.

### Experiment 1: Sex Facilitation

Sprague Dawley male rats mated in their home cages for 5 consecutive, daily mating sessions. Animals were divided into 2 experimental groups and were tested for sexual behavior either 1 week or 1 month following after last mating session (Test Day; n = 7 per group). Groups were matched on parameters of sexual behavior and no significant differences were detected between groups for any sexual behavior measure during any mating session while gaining sexual experience.

#### Data Analysis

Within group comparisons were made assessing Day 1 and 5 of sexual experience to determine facilitation of sexual behavior with sexual experience, between Day 1 and Test day, and between Day 5 and Test day (either 1 week or 1 month after Day 5) for mount, intromission and ejaculation latencies using Wilcoxin Signed Rank test with significance level of 5%.

### Experiment 2: Ionotropic Glutamate Receptor Subunit Redistribution/Expression

To examine ionotropic receptor redistribution, a paradigm similar to experiment 1 was utilized. Sexually naive male Sprague Dawley rats were divided into sexually experienced and naïve groups. For the sexually experienced groups, sexual experience was gained through 5 consecutive, daily mating sessions (as described above). The sexually experienced males were then divided into 3 experimental groups (matched for sexual behavior parameters) for tissue collection, either 1 day, 1 week, or 1 month following last mating session. Brains from sexually naïve controls (handled as described above) were collected at identical time points after final handling. The groups included: sexually experienced (AMPAR: 1D, n = 9; 1W, n = 12; 1M, n = 12; NMDAR: 1D, n = 9, 1W or 1M, n = 6) or sexually naïve (AMPAR: 1D, n = 9; 1W, n = 12; 1M, n = 12; NMDAR: 1D, n = 9, 1W or 1M, n = 6).

#### Surface Receptor Cross-Linking

Animals were euthanized with sodium pentobarbital (270 mg/kg; i.p.) followed by decapitation. Following decapitation, each brain was rapidly removed and immediately placed into ice-cold saline. Bilateral NAc was dissected using a rat brain matrix (ASI Instruments, Warren, MI, USA) and scalpel blade according to NAc boundaries defined by Paxinos & Watson (1998). Next, NAc tissue was chopped into 400×400 µm cubes using a McIlwain tissue chopper (Vibratome, St. Louis, MO, USA). Methodology for AMPA and NMDA receptor subunit cross-linking were based on Boudreau & Wolf [53]. Immediately following chopping, brain tissue was rapidly transferred to an Eppendorf tube containing 1 mL of ice-cold aCSF spiked with the protein cross-linking reagent bis(sulfosuccinimidyl)suberate (BS^3^, 2 mM; Pierce Biotechnology, Rockford, IL, USA) and incubated for 30 minutes on a rocker at 4°C. BS^3^ does not cross cell membranes, enabling it to selectively cross-link surface-expressed proteins with sulfide bonds, thus forming high molecular weight aggregates, while intracellular proteins remain unmodified. This reaction enables surface and intracellular pools of protein to be distinguished based on MW using sodium dodecyl sulfate-polyacrylamide gel electrophoresis (SDS-PAGE) and Western Blot analysis. The cross-linking reaction was quenched by the addition of 100 µL of 1 M glycine for 10 min at 4°C. Tissue was pelleted by centrifugation at 14 000 rpm for 2 minutes at 4°C and the supernatant was discarded. The pellets were resuspended in 400 µL of ice-cold lysis buffer [25 mM Hepes (pH 7.4), 500 mM NaCl, 2 mM EDTA, 1 mM DTT, 1 mM PMSF, 20 mM NaF, 0.1% Nonidet P-40 (0.1%), protease inhibitor cocktail (Ministop, Roche Diagnostics GmbH, Mannheim, Germany) and 1× phosphatase inhibitor cocktail (Phosstop, Roche Diagnostics GmbH,)]. Samples were sonicated for 5 seconds to disrupt tissue, then centrifuged at 14 000 rpm for 2 min at 4°C and immediately placed back into ice block. Supernatant was transferred to a new Eppendorf tube, from which 30 µL of sample were put on ice and remaining supernatant was stored at −80°C for Western Blot analysis. For each sample the protein concentration of cross-linked lysates was determined using a BCA assay (ThermoFisher Scientific Inc., Waltham, MA) and NanoDrop ND-1000 spectrophotometer (ThermoFisher Scientific Inc., Waltham, MA).

#### Western Blot Analysis

Protein samples (20 µg) were loaded and electrophoresed on 4–15% gradient Tris-HCl gels (Bio-Rad Laboratories Ltd., Mississauga, Ontario, Canada) using a Mini Trans-Blot Cell system (Bio-Rad Laboratories Ltd.) and Tris-Glycine-SDS running buffer [25 mM Tris, 192 mM Glycine, 0.1% SDS (pH8.3)]. Precision Plus protein All Blue standards (Bio-Rad Laboratories Ltd.) were used as molecular weight markers. Following separation, proteins were transferred to Millipore Immobilon-FL polyvinylidene difluoride membranes (PVDF; Millipore, Billerica, MA, USA) using the Trans-Blot Cell wet Blotting system (Bio-Rad Laboratories Ltd.) for immunoblotting. The protein transfer was run in transfer buffer (20% methanol and 0.037% SDS in Tris-Glycine [25 mM Tris, 192 mM Glycine (pH 8.3)] at 82 V for 1 hr at room temperature (RT). All samples were run in at least duplicate, balanced across groups, and across individual gels.

Next, membranes were incubated in a 2∶3 solution of Odyssey Blocking Buffer (LI-COR Biosciences, Lincoln, NE) and Tris-Buffered Saline (TBS; 50 mM Tris and 150 mM NaCl (pH8.0)) for 1 hr on a shaker tray at RT. Membranes were then individually incubated for 16 hrs on a shaker at 4°C with either rabbit polyclonal anti-GluA1 (∼106 kDa; 1∶1K; Millipore, Cat # AB1504) and GluA2 (∼100 kDa; 1∶4K; Millipore, Cat # AB1768), or mouse monoclonal anti-NR1 (∼130 kDa; 1∶2K; Upstate (Millipore), Cat # 05-432). These primary antibodies have been previously used and validated [84], [85], and produced a single band at the appropriate molecular weight that was prevented by preabsorption of the antibody with peptide when used on non cross-linked tissue. All antibodies were diluted in a 2∶3 mix of Odyssey Blocking Buffer with TBS-T (TBS+0.05% Tween-20 (pH8.0). Following three 10-min washes in TBS-T, the membranes were incubated in secondary antibodies diluted in a 2∶3 mix of Odyssey Blocking Buffer and TBS-T for 1 hr at RT. The secondary antibodies included Alexa-680 conjugated goat anti-rabbit (1∶5K; Invitrogen, Carlsbad, CA, USA) or IR Dye800 CW-conjugated goat anti-mouse (1∶10K; LI-COR Biosciences). Fluorescent immunoreactivity was visualized and images captured using an Odyssey 2.1 scanner (LI-COR Biosciences).

#### Quantification and statistical analyses

For each protein sample, fluorescence intensity levels for each band (High MW, >250 kDa for surface expression, and bands at the specific MW listed above for intracellular expression) were determined using the Odyssey software and averages were calculated for each animal. Surface (S), intracellular (I), ratio (S/I, measure of receptor subunit distribution) and total (S+I, measure of total receptor subunit expression) band density values were normalized to the mean values of corresponding sexually naïve control groups. There was no difference in fluorescence intensity between the replicates of each sample (loaded in different gels), confirming the lack of variability in the loading of the protein samples. All Western Blot data was analyzed between sexually experienced and sexually naïve controls at the same time point using unpaired t-tests with a significance level of 0.05

### Experiment 3: Electrophysiology

The same experimental design was used as described above for experiments 1 and 2. Sexually experienced males were divided into 3 experimental groups, matched for sex behavior performance, and based on the time of tissue collection 1 day, 1 week or 1 month following last mating session (1 day, n = 7; 1 week, n = 9; 1 month, n = 10). Sexual behavior in these younger male rats did not differ from the sexual behavior of the older male rats in experiments 1 and 2. Rats were anaesthesized with sodium pentobarbital (270 mg/kg; i.p) followed by transcardial perfusion with ice-cold oxygenated (95% O_2_, 5% CO_2_) preparation solution containing [250 mM Sucrose, 2.5 mM KCl, 1.25 mM NaH_2_PO_4_, 4 mM MgCl_2_, 0.1 mM CaCl_2_, 26 mM NaHCO_3_, 10 mM glucose, 3 mM myoinositol, 2 mM sodium pyruvate, and 0.5 mM ascorbat]. The brain was excised and placed in ice-cold oxygenated preparation solution. Sagittal brain slices, 400 µm thick, were obtained from each animal using a vibratome (Microm, Walldorf, Germany). A total of 4 slices per brain were transferred to a holding chamber with artificial cerebrospinal fluid (aCSF) [125 mM NaCl, 2.5 mM KCl, 1.25 mM NaH_2_PO_4_, 1.3 mM MgCl_2_, 2.5 mM CaCl_2_, 26.2 mM NaHCO_3_ and 10 mM glucose], heated to 32°C for 30 min, and then allowed to recover to room temperature for at least 1 hr before a single slice was placed in a recording chamber. This chamber was superfused with oxygenated aCSF at 22°C. There were no differences between groups for any sexual behavioral parameters. Brains were collected from sexually naïve controls at each of the 3 time points after final handling (n = 3–4 each).

Shell neurons were examined in medial NAc slices that did not contain dorsal striatum [72]. Stainless steel bipolar microelectrodes were placed in the prelimbic cortex-NAc border rostrally to the recording electrode for presynaptic stimulation of cortical afferent fibers. Vital medium spiny neurons were visually identified by their soma size (around 30–35 µ M diameter) and a relatively negative resting membrane potential fo −75 to −85 mV. Recordings were made using an Axopatch 200A amplifier and a sampling rate of 20 kHz (Digidata 1340), with a 10 kHz low-pass filter. PClamp 9.0 was used for experiment protocols and analysis. Afferents were stimulated with paired pulses (50 ms ISI) at 0.1 Hz and neurons using a bipolar concentric tungsten electrode. Cells were voltage-clamped at −80 mV, and depolarized to +40 mV for 500 ms before synaptic stimulation, in order to relieve the magnesium block of NMDA currents. AMPA/NMDA ratios were determined by taking the average of EPSCs at +40 mV in the absence or presence of the NMDAR antagonist AP5 (50 µM; whole cell currents 30× control and 30× in the presence of AP5). NMDA response was calculated by subtracting AP5 recordings from control. The peak of AMPAR EPSC was divided by the peak of the NMDAR EPSC to yield an AMPA/NMDA ratio. In order to determine paired pulse ratios, measurements were taken at −80 mV with presynaptic stimulation (30×).

#### Data Analysis

Data from the sexually naïve controls were combined to form one control group (n = 10 neurons in 10 animals) since no statistical differences were detected between time points within controls. Sexually experienced groups (1 day, n = 7; 1 week, n = 9; 1 month, n = 10 neurons; typically one neuron per animal) were compared to the sexually naïve control group using a one-way ANOVA (factors: time interval) followed by a Fisher LSD for *post hoc* comparisons with a significance level of 5%.
